# Oncogenic and Immunological Roles of FRS2 and its Potential Value in Retroperitoneal Liposarcoma: from Bioinformatics Analysis to Clinicopathological Evidence

**DOI:** 10.7150/ijms.103802

**Published:** 2025-03-10

**Authors:** Hao Yu, Shuquan Li, Yifan Wu, Zhen Wang, Xiaopeng Wang, Sha Zhang, Xiaoya Guan, Bin Dong, Chunyi Hao, Xiuyun Tian, Ang Lv

**Affiliations:** 1 Key Laboratory of Carcinogenesis and Translational Research (Ministry of Education/Beijing), Department of Hepato-Pancreato-Biliary Surgery, Peking University Cancer Hospital and Institute, Beijing, China.; 2 Department of Critical Care Unit, Shandong Provincial Hospital Affiliated to Shandong First Medical University, Jinan, Shandong, China.; 3 Key Laboratory of Carcinogenesis and Translational Research (Ministry of Education), Center Laboratory, Peking University Cancer Hospital & Institute, Beijing, China.

**Keywords:** FRS2, retroperitoneal liposarcoma, therapeutic target, immunotherapy, prognosis, survival analysis

## Abstract

**Background:** Retroperitoneal liposarcoma (RLPS) is a rare malignancy with no effective treatment beyond surgical intervention. Identifying novel therapeutic targets and prognostic markers is critical to improving outcomes. Fibroblast growth factor receptor substrate 2 (FRS2), located near MDM2 on chromosome 12q13-15, has a biological role and prognostic value in liposarcoma, which remain to be fully explored.

**Methods:** Bioinformatics tools were used to analyze the differential expression of FRS2 across various malignancies using public databases, such as GTEx, TCGA, and cBioPortal. In sarcomas (SARC), clinicopathological features, prognostic outcomes, co-expressed genes, levels of tumor-infiltrating immune cells, immunostimulators, major histocompatibility complex (MHC) molecules, and immunochemokines were extracted from multiple public databases. Tumor specimens from 82 RLPS patients at our sarcoma center were collected, and FRS2 expression was assessed through immunohistochemistry.

**Results:** FRS2 was found to be upregulated and amplified in most cancers. GEPIA 2 analysis showed significant variation in FRS2 mRNA expression across cancer types, especially in sarcomas (SARC). Lower FRS2 expression in SARC was correlated with improved overall survival (OS) and disease-free survival (DFS). FRS2 may affect the tumor immune microenvironment, inhibiting immune cell infiltration and promoting immune evasion. In our RLPS cohort, FRS2 overexpression was observed in 58.53% (48/82) of cases and was correlated with age (P = 0.009). High FRS2 expression was associated with poorer OS and DFS (P = 0.049 and P < 0.001, respectively), and multivariate analysis confirmed FRS2 as an independent prognostic factor.

**Conclusion:** FRS2 may serve as a potential prognostic biomarker and therapeutic oncogene target. Additionally, FRS2 could play a role in immune cell infiltration in SARC and represents a promising immunotherapeutic target for cancer treatment.

## Introduction

Liposarcoma (LPS) is a malignant mesenchymal tumor that constitutes approximately 15-20% of all soft tissue sarcomas (STS) and is one of the most common STS subtypes 1-2. LPS is categorized into five subtypes: well-differentiated liposarcoma (WDLPS), dedifferentiated liposarcoma (DDLPS), myxoid/round cell liposarcoma (MLPS), pleomorphic liposarcoma (PLPS), and myxoid pleomorphic liposarcoma (MPLPS) 2. In the retroperitoneum, WDLPS and DDLPS are the most frequently occurring types of STS 3. Currently, surgical resection remains the sole treatment option for retroperitoneal liposarcoma (RLPS) 4. However, achieving a complete resection of RLPS is challenging due to the tumor's location and propensity for local infiltration, resulting in a high incidence of local recurrence 5. Incomplete resection and disease recurrence are associated with poor prognosis 6. Moreover, there are limited effective treatment options for locally advanced and disseminated RLPS 7. Consequently, there is a need to identify new therapeutic targets and prognostic biomarkers to enhance treatment strategies and improve outcomes in RLPS.

Amplification of the MDM2 and CDK4 genes on chromosome 12q13-15 is commonly observed in WDLPS and DDLPS 8. This amplicon spans several megabases and includes multiple genes. Such discontinuous amplification can result in diverse patterns of protein overexpression, which may confer varying growth advantages to cancers among different patients 9. The FRS2 gene, located near MDM2 on chromosome 12q13-15, encodes fibroblast growth factor receptor substrate 2 (FRS2), a member of the adaptor/scaffold protein family 10. FRS2 is a critical mediator in the fibroblast growth factor receptor (FGFR) signaling pathway, binding to FGFR through its phosphotyrosine-binding domain and activating downstream signaling cascades 11. Previous studies have demonstrated that aberrant activation or amplification of FRS2 is linked to tumorigenesis in thyroid cancer, prostate cancer, and high-grade serous ovarian cancer 12. These findings suggest that FRS2 may be a promising target for therapeutic intervention in liposarcomas.

In this study, we utilized publicly available data to investigate the correlation between FRS2 expression and prognosis, aiming to clarify the prognostic significance and related biological functions of FRS2 in sarcoma. Along with performing enrichment analysis of FRS2, we explored the relationship between FRS2 expression and the tumor-immune microenvironment (TIME). Additionally, we assessed FRS2 expression in our own retroperitoneal liposarcoma (RLPS) samples, examining its clinicopathological relevance and prognostic value within our patient cohort.

## Materials and Methods

### Genetic alteration analysis of FRS2

The cBioPortal platform (https://www.cbioportal.org/) was utilized to search for genetic alteration information related to FRS2. All TCGA Pan-Cancer Atlas studies were included in this analysis. Somatic mutation frequency and genomic information of FRS2 mutations across various cancers were explored using the ”Cancer Types Summary and Mutations” and “mRNA vs. Study” modules. Mutation sites were obtained from the “mutations” module (data retrieved on 2023-12-15).

### Genes expression and datasets obtained

The TCGA (https://cancergenome.nih.gov) and GTEx (https://gtexportal.org/) databases were used to obtain FRS2 mRNA expression data from tumor samples, corresponding adjacent non-cancerous samples, and normal samples. The 33 cancer types examined are listed in Supplemental Table 1 (data retrieved from databases on 2023-12-15).

### GEPIA2 analysis

We utilized the Gene Expression Profiling Interactive Analysis 2 (GEPIA2) tool to assess FRS2 expression in tumor tissues versus their corresponding normal tissues, using data from TCGA and GTEx cohorts. The “Expression DIY” kit from the “Expression Analysis” module was employed to analyze differential expression of FRS2 across various tumor samples with parameters set to p-value “0.05,” matched normal data from both TCGA and GTEx, and all cancer types. Additionally, GEPIA2 was used to examine the relationship between MDM2 and FRS2 in sarcoma (SARC) (data retrieved from databases on 2023-12-15).

### Survival analysis in sarcomas

The potential prognostic value of FRS2 in sarcoma was assessed using overall survival (OS) and disease-free survival (DFS) data from the TCGA database (data retrieved from databases on 2023-12-15).

### Immunological correlation analysis

The Tumor Immune Estimation Resource (TIMER) (http://cistrome.org/TIMER/) was utilized to infer the abundance of tumor-infiltrating immune cells based on gene expression profiles. Clinical relevance of immune subsets was analyzed with consideration of covariates such as age, gender, ethnicity, and tumor stage. Additionally, the TISIDB database (http://cis.hku.hk/TISIDB/) was used to analyze the correlation between FRS2 expression and the abundance of tumor-infiltrating lymphocytes (TILs), as well as its relationship with three types of immunomodulators, chemokines/receptors and immune subtypes (data retrieved from databases on 2023-12-15).

### PPI network construction and screening of core network genes

The Protein-Protein Interaction (PPI) network was constructed using the STRING database (https://stringdb.org/). This database helps analyze differential genes identified from various expression groups. For constructing the PPI network, we selected interactions with a minimum score of 0.9. Core nodes, crucial for network stability, were identified, and a bar chart was created in R to visualize the number of core nodes. The 10 genes with the highest number of adjacent nodes were designated as the core genes in this network analysis (data retrieved on 2023-12-15).

### Clinical patients and samples

All tumor samples in this study were collected from 82 RLPS patients who underwent surgical resection at the Sarcoma Centre of the Peking University Cancer Hospital (Beijing, China) between March 2009 and August 2017. Clinicopathological features and follow-up data were recorded. RLPS cases were classified as either well-differentiated liposarcoma (WDLPS) or dedifferentiated liposarcoma (DDLPS) based on the World Health Organization classification and graded according to the Fédération Nationale des Centers de Lutte Contre le Cancer (FNCLCC) grading system 13. Among the 82 cases, 56 were diagnosed with dedifferentiated liposarcoma (DDLPS) and 26 with well-differentiated liposarcoma (WDLPS). Patients were followed up from June 2013 to October 2019, with a median follow-up time of 35.1 months (range: 2.3-130.5 months). The average age of the patients was 58.2 ± 9.7 years. Detailed characteristics are outlined in Supplemental Table 2. Patients who had received chemotherapy or radiotherapy prior to surgery were excluded from the study. Written informed consent was obtained from all participants. This study was approved by the Institutional Review Board of the Cancer Hospital of Peking University (approval number 2019KT19).

### Immunohistochemical (IHC) assessment and staining evaluation

IHC was performed following standard protocols. The antibody used was anti-FRS2 (1:500, Abcam, #ab137458). Two independent pathologists confirmed the results. Staining intensity was categorized as "-", "+", "++", and "+++". "-" indicated negative staining, while "+", "++", and "+++" indicated positive staining. For subsequent analyses, "-" and "+" were classified as low expression, whereas "++" and "+++" were classified as high expression.

### Statistical analysis

The χ2 test and Fisher's exact test were employed to evaluate correlations between immunohistochemical staining and clinicopathological parameters. Survival curves were generated using the Kaplan-Meier method, and the association between FRS2 expression and prognosis was assessed using the log-rank test. Results were considered statistically significant if the *P* value was < 0.05. Statistical analyses were conducted using SPSS software (version 26.0; Chicago, IL, USA).

## Results

### Genetic alteration of FRS2 in pan-cancers

**Figure 1:**
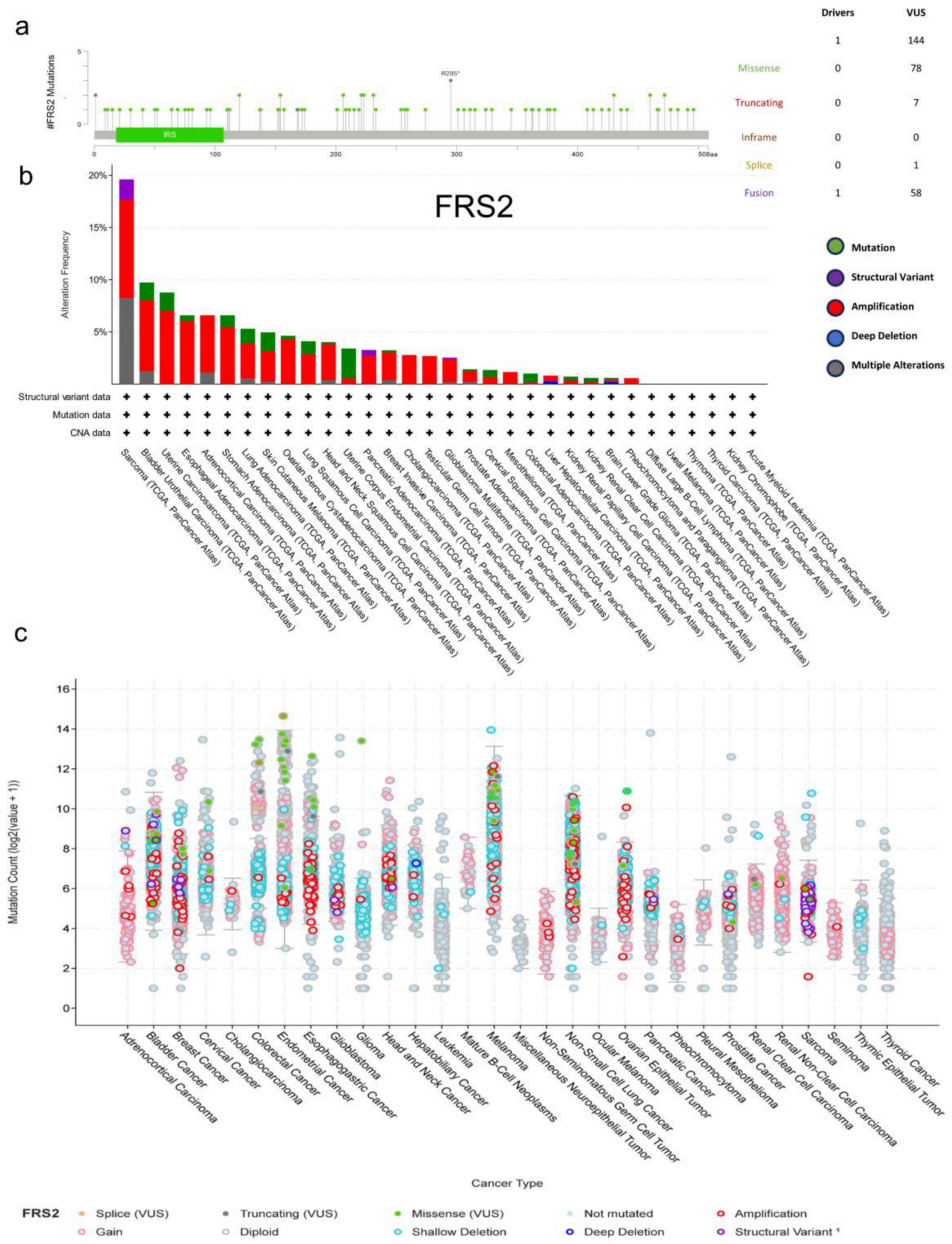
** Genetic alteration of FRS2 in pan-cancers. (a)** Mutation diagram of FRS2 across protein domains; **(b)** Bar chart of FRS2 mutations in 32 cancer studies based on TCGA Pan-Cancer Atlas Studies; **(c)** Mutation counts and types of FRS2 in 32 cancers.

The cBioPortal online tool was utilized to analyze genetic variations in FRS2 expression across cancer types. This analysis included all TCGA Pan-Cancer Atlas studies, comprising 32 studies and a total of 10,967 samples. We identified 144 mutation sites within amino acids 0 to 508, which included 78 missense mutations, 7 truncating mutations, 1 splice mutation, and 58 structural variants/fusions. Among these, R295 was found to be the most frequent mutation site in FRS2 (Figure 1a). The most prevalent mutation types observed were missense and fusion mutations, with FRS2 mutations being most commonly found in sarcoma (Figure 1b). Among the 32 cancer types analyzed, amplification mutations, gain mutations, and shallow deletions were frequently associated with FRS2 mRNA expression across different cancers (Figure 1c). Additionally, Supplementary Figure 1 indicated a positive correlation between FRS2 and MDM2 expression in sarcoma (SARC), with a correlation coefficient of R = 0.63 and a P value of < 0.001.

### Relationship between FRS2 expression and prognosis in SARC

**Figure 2:**
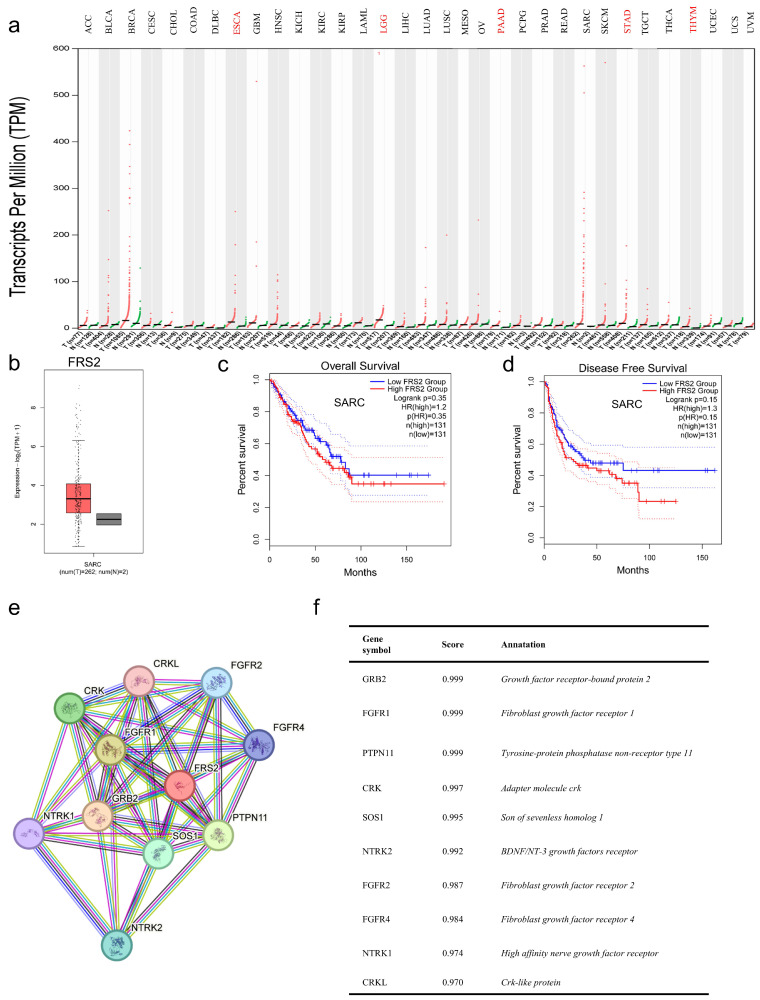
** FRS2 expression analysis. (a)** Comparison of FRS2 expression between tumor and paired normal samples based on the TCGA database; **(b)** The expression of FRS2 in SARC based on TCGA database; **(c)** OS and **(d)** DFS of FRS2 in SARC using the Kaplan-Meier analysis; **(e)** The PPI network of FRS2; **(f)** Annotation of FRS2-interacting proteins and their coexpression scores.

To further investigate the expression levels of FRS2, we analyzed tumor datasets from the TCGA database alongside corresponding normal tissues from the GTEx cohort using GEPIA2. We observed a significant increase in FRS2 expression across most cancer types in paired tumor samples compared to their corresponding normal samples (Figure 2a). Specifically, FRS2 mRNA expression was elevated in sarcoma (SARC) (Figure 2b). Next, we assessed the prognostic value of FRS2 in SARC using Kaplan-Meier analysis. As illustrated in Figure 2c, patients in the low FRS2 expression group showed better OS; however, this correlation was not statistically significant (P = 0.35). Similarly, for DFS, lower FRS2 expression was associated with better prognosis, but again, the difference was not significant (P = 0.15, Figure 2d). To understand the progression of malignancy and its underlying molecular mechanisms, we analyzed the PPI network of FRS2 proteins using the STRING tool. Figures 2e and 2f present the top 10 proteins associated with FRS2, along with their corresponding gene names, scores, and gene annotations. These proteins include GRB2, FGFR1, PTPN11, CRK, SOS1, NTRK2, FGFR2, FGFR4, NTRK1, and CRKL.

### Immune-related characteristics of FRS2 in SARC

**Figure 3:**
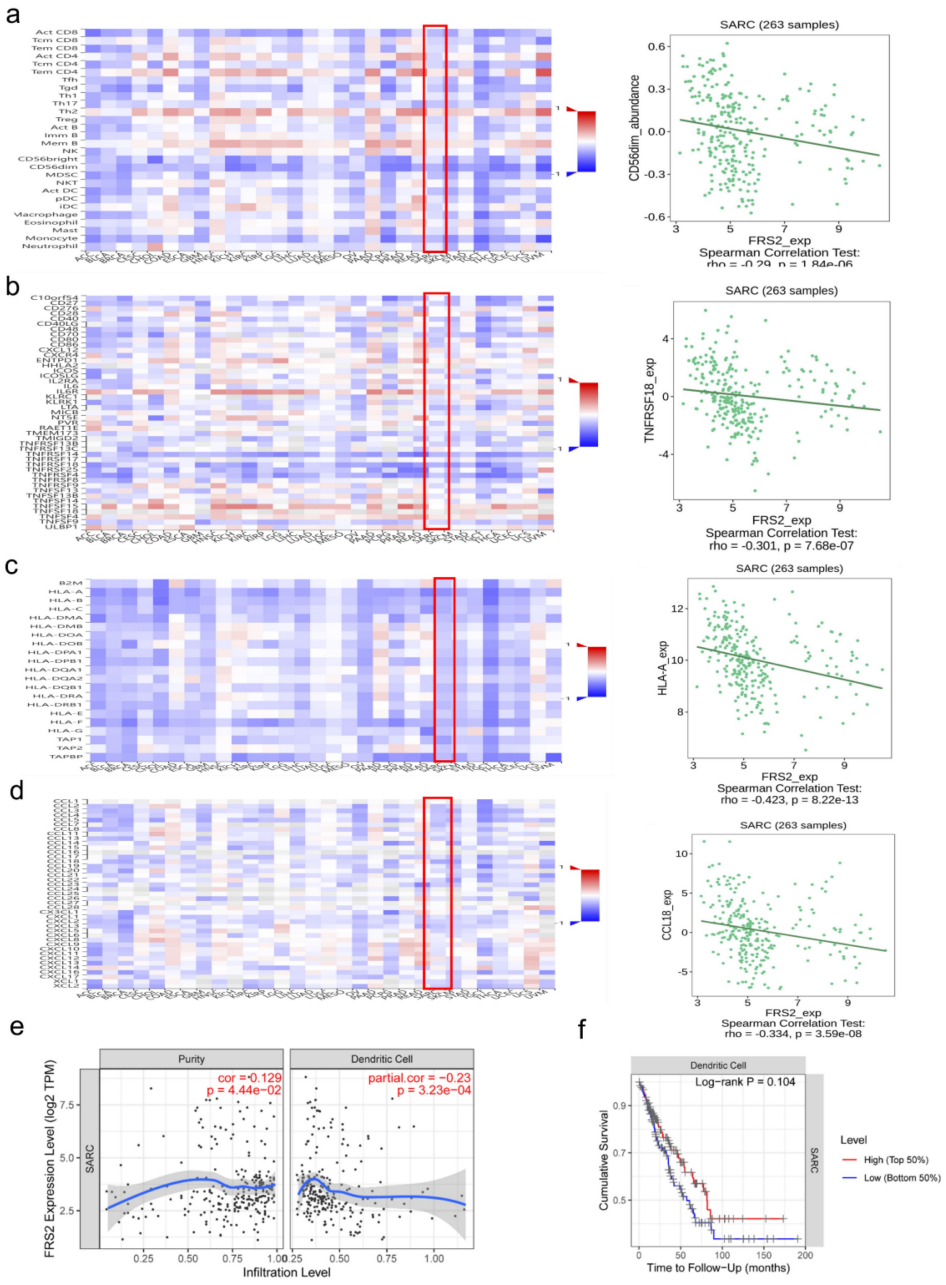
** Correlation analysis of FRS2 expression with immune-related characteristics in SARC. (a)** Heatmap analysis of the correlation between FRS2 and tumor-infiltrating lymphocytes (TILs) in tumors, FRS2 in SARC is positively correlated with CD56dim natural killer cell. **(b)** Heatmap analysis of the correlation between FRS2 and immunostimulatory in tumors, FRS2 in SARC is positively correlated with TNFRSF18. **(c)** Heatmap analysis of the correlation between FRS2 and MHC molecules in tumors, FRS2 in SARC is positively correlated with HLA-A. **(d)** Heatmap analysis of the correlation between FRS2 and immunochemokines in tumors, FRS2 in SARC is positively correlated with CCL18. **(e)** Scatter plot of correlation between FRS2 and dendritic cell infiltration in SARC. **(f)** The relationship between dendritic cell infiltration and survival based on TIMER database.

The tumor immune microenvironment (TIME) plays a crucial role in tumor progression and response to treatment. TIME encompasses various immune cells, cytokines, and other factors that influence tumor behavior and therapeutic outcomes. Understanding TIME is crucial for developing targeted therapies and improve treatment strategies 14. Immune cell infiltration in the tumor microenvironment significantly impacts immunotherapy efficacy and patient prognosis 15. We utilized the TISIDB and TIMER databases to identify significant immune-related characteristics associated with FRS2 in sarcoma (SARC) (P < 0.05). The TISIDB database was used to analyze the relationship between FRS2 expression and the abundance of tumor-infiltrating lymphocytes (TILs), two types of immunomodulators, and chemokines. Spearman analysis was performed to assess the correlations between FRS2 expression and immune-related characteristics across SARC. Our findings indicated that FRS2 expression was negatively correlated with most TILs (red frame), with CD56dim natural killer cells showing the most significant difference among TILs (R = -0.29, P < 0.0001, Figure 3a). It is known that the clinical efficacy of several chemotherapies involves the stimulation of anti-cancer immunity 16. FRS2 expression was negatively correlated with most immunostimulators (red frame), with TNFRSF18 showing the most significant difference among these (R = -0.301, P < 0.0001, Figure 3b). Major histocompatibility complexes (MHCs) in the tumor immune microenvironment play a key role in antigen presentation and T cell receptor recognition, affecting the efficacy of immunotherapy 17. FRS2 was found to inhibit MHC-mediated tumor immune antigen presentation (red frame), with HLA-A being the most significantly affected MHC molecule (R = -0.423, P < 0.001, Figure 3c). CCL18 was identified as the most significantly different immunochemokine, with FRS2 inhibiting almost all immunochemokines (R = -0.334, P < 0.001, Figure 3d). We utilized the TIMER database to evaluate the relationship between FRS2 expression and immune cell infiltration. Our analysis revealed that FRS2 was associated with dendritic cells (Rho = -0.23, P < 0.001) in sarcoma (SARC) (Figure 3e). Notably, higher levels of dendritic cells were associated with better survival, although this difference was not statistically significant (P = 0.104, Figure 3f). Additionally, based on the TIMER database, we examined the relationship between gene expression levels and immune cell infiltration after adjusting for purity (R = 0.129, P < 0.05, Figure 3e). Tumor purity refers to the proportion of tumor cells within the tumor tissue, and purity adjustment aids in objectively analyzing tumor samples while minimizing analysis bias 18. FRS2 influenced dendritic cell immune infiltration, and dendritic cells impacted survival prognosis in SARC (Figure 3f). These findings suggest that targeting FRS2 could be a promising approach for sarcoma immunotherapy.

### Correlations of FRS2 expression levels with survival of RLPS patients

**Figure 4:**
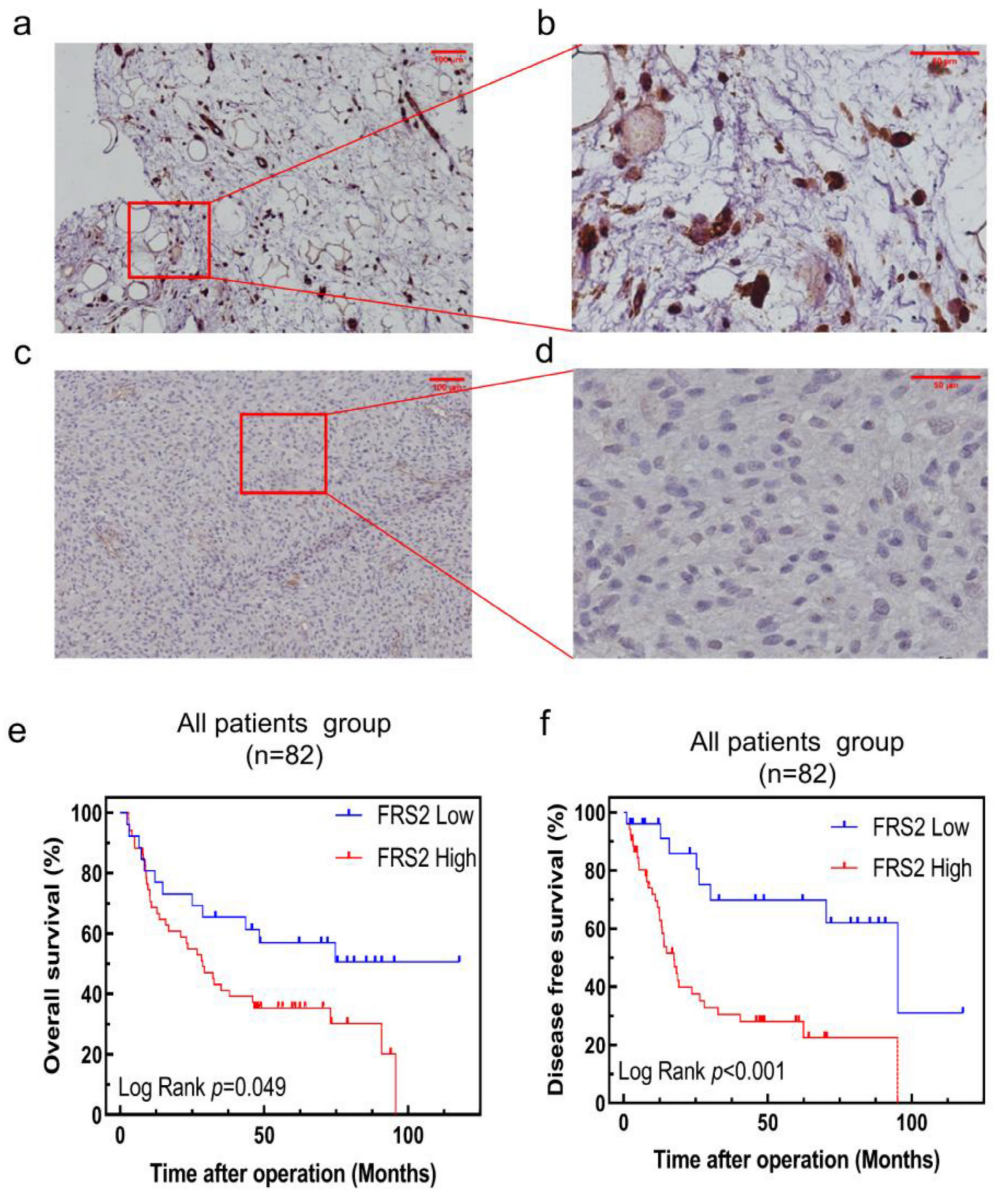
** FRS2 protein expression in RLPS by immunohistochemistry and Correlation between FRS2 expression levels and prognosis of patients with retroperitoneal liposarcoma. (a)** positive FRS2 expression. (scale bar 100μm); **(b)** positive FRS2 expression (red frame area, scale bar 50μm); **(c)** negative FRS2 expression (scale bar 100μm); **(d)** negative FRS2 expression (red frame area, scale bar 50μm). FRS2 were typically located in the cytoplasm. Kaplan-Meier survival curves for **(e)** OS in 82 patients; **(f)** DFS in 82 patients.

To further validate the clinical significance and prognostic role of FRS2 in RLPS, we conducted a retrospective study with a real-world cohort. FRS2 expression was evaluated by immunohistochemistry (IHC) in 82 patients. Figure 4a-4d displays typical IHC staining for FRS2 proteins, which were primarily located in the cytoplasm and on the membrane. In our cohort of 82 RLPS patients, we validated that high FRS2 expression was significantly associated with poorer prognosis. Kaplan-Meier analysis of OS and DFS is shown in Figure 4e-4f. High FRS2 expression correlated with poorer OS. For all 82 patients, median OS was 35.0 ± 27.3 months for high FRS2 versus 50.7 ± 34.1 months for low FRS2 (P = 0.049, Figure 4e). High FRS2 expression was linked to poorer DFS among all patients (P = 0.0006). The median DFS was 23.1 ± 22.9 months versus 45.4 ± 35.7 months for all 82 patients.

Out of the 82 specimens collected from our center, 48 (58.53%) showed high FRS2 expression. Specifically, 32 of 56 DDLPS cases (57.14%) and 16 of 26 WDLPS cases (61.54%) had high FRS2 expression (Supplemental Table 3). We then evaluated the correlation between FRS2 expression and clinicopathological features in RLPS. High FRS2 expression was significantly associated with age (P = 0.009, Supplemental Table 3). However, no significant relationships were found between FRS2 expression and gender, tumor size, FNCLCC grade, histopathological classification, multifocality, necrosis, or recurrence.

We assessed the prognostic value of FRS2 in RLPS patients using univariate and multivariate Cox regression analyses, with P < 0.05 considered significant due to the limited case number. In the univariate analysis, FRS2 expression was not found to be a significant predictor of OS (hazard ratio (HR): 1.915; 95% confidence interval (CI): 0.990-3.702; P = 0.053; Table 1). Additionally, FNCLCC grade (P = 0.002), histology (P = 0.005), and recurrence (P = 0.013) were significant predictors in the univariate analysis (Table 1). In the multivariate Cox regression analysis, recurrence emerged as an independent prognostic factor for OS in RLPS patients (P = 0.025, Table 1).

In the univariate and multivariate Cox regression analyses, we identified that FRS2 expression was a significant predictor of DFS (hazard ratio (HR): 3.911; 95% confidence interval (CI): 1.707-8.957; P = 0.001; Table 2). Additionally, tumor size (P = 0.056), FNCLCC grade (P = 0.015), histology (P = 0.004), and necrosis (P = 0.057) were significant predictors in the univariate analysis (Table 2). For the multivariate Cox regression analysis, we adjusted for the statistically significant features identified in the univariate analysis. The results indicated that FRS2 expression are independent prognostic factors for OS in RLPS patients (P = 0.001 for FRS2 expression; Table 2).

## Discussion

RLPS is a rare malignancy for which surgical resection remains the primary treatment option. For recurrent and advanced cases, treatment options are limited, highlighting the urgent need for new targeted therapies. Notably, more than 50% of liposarcomas are WDLPS and DDLPS 19-22. Both subtypes are characterized by large marker chromosomes with amplification of the 12q13-15 chromosomal region, leading to increased MDM2 copy number 20. This chromosomal region is complex and contains several critical genes that remain underexplored. Among these, the FRS2 gene, located within 12q13-15 near MDM2 and CDK4, is particularly noteworthy due to its relatively under-investigated role in RLPS 21.

FRS2 is an adaptor protein that interacts with receptor tyrosine kinases (RTKs) such as FGFR, neurotrophin receptor, RET, and ALK, facilitating signal transduction from FGFRs 12, 26. Our analysis using TCGA data confirms a positive correlation between MDM2 and FRS2 expression (R = 0.63, P < 0.01). Amplification of FRS2 in liposarcoma has been documented 25, and we aim to evaluate its prognostic value and potential as a novel therapeutic target for TKI-based treatments. Additionally, we explored the impact of FRS2 on tumor immunity, offering new perspectives for immunotherapy in RLPS.

This study identified the most common types of FRS2 mutations and assessed FRS2 mRNA expression across various cancers. We explored the prognostic value of FRS2 expression in SARC and constructed PPI and mRNA regulatory networks for FRS2. We then investigated how FRS2 expression correlates with the tumor immune microenvironment and immune cell infiltration in SARC. Additionally, we examined the potential of FRS2 as a target for immunotherapy and analyzed its correlation with prognosis in our cohort of RLPS patients.

FRS2 amplification is common across 32 cancer types, with nearly 20% of SARC population exhibiting FRS2 amplification, the highest prevalence among all cancer types. FRS2 expression levels were found to be elevated in various tumors, including CHOL, ESCA, HNSC, LIHC, and STAD, as well as in SARC. This elevation may be linked to other forms of genetic alterations, such as mutations, structural variants, and deep deletions observed in different cancers. Notably, in SARC, FRS2 mutations were predominantly amplifications, aligning with the finding that FRS2 mRNA expression in SARC samples was mainly driven by gains and amplifications.

Although FRS2 is not widely expressed in normal tissues, TCGA and GTEx data revealed significantly higher expression levels in most cancers compared to corresponding normal tissues. In our study, we evaluated the prognostic value of FRS2 in SARC using Kaplan-Meier analysis. While lower FRS2 expression was associated with better prognosis for both OS and DFS, the differences between high and low expression groups were not statistically significant. Previous studies have shown that FRS2 is linked to poorer prognosis in various cancers. For instance, high FRS2 expression in bladder tumors has been shown to impair endothelial cell recruitment and tube formation, contributing to adverse outcomes 22. FRS2 amplifications have been associated with very poor prognosis and atypical clinical features in neuroblastoma patients, indicating that FRS2 is not only a prognostic marker but also a potential therapeutic target 23. In small cell lung cancer (SCLC), FGFR inhibitors demonstrated delayed progression in both *in vivo* and *in vitro* experiments by inhibiting downstream MAPK and PI3K-Akt signaling pathways through the inhibition of FRS2 phosphorylation 24. In the *in vivo* and *in vitro* experiments with ovarian cancer, FRS2 inhibitors prevented the activation of FRS2 and interrupted the FGFR signaling pathway, thereby inhibiting tumor invasion and growth 25. In the *in vivo* and *in vitro* experiments with bone metastatic tumors, FRS2 expression elevation is positively correlated with increased angiogenesis and poor prognosis. Furthermore, it could serve as an independent prognostic marker for patients with bone metastatic tumors. Experimental results indicate that reducing FRS2 expression in bone metastatic tumor cells can reduce the proliferation, migration, and angiogenesis of endothelial cells. Further studies show that miR-429 and miR-206 inhibit FRS2 expression, making them promising therapeutic candidates for anti-angiogenic treatment of bone metastatic tumors 26. Consistent with pan-cancer analyses, our study found high FRS2 expression in 65.9% (54/82) of retroperitoneal liposarcoma (RLPS) specimens. Furthermore, we established that high FRS2 expression serves as an independent predictive factor for both OS and DFS in these patients.

High FRS2 expression was correlated with worse DFS and OS in our cohort of 82 RLPS patients, although this association was not significant in the broader TCGA dataset of 259 sarcomas. This discrepancy may reflect FRS2's predictive value in specific sarcoma subtypes. Elevated FRS2 levels in RLPS suggest its potential as a therapeutic target. Previous studies have also associated FRS2 overexpression with increased angiogenesis and poorer prognosis in osteosarcoma 26. Thus, further studies are needed to clarify FRS2's role in other cancers. Additionally, our study found a significant association between high FRS2 expression and age (P=0.009), suggesting that FRS2 may influence RLPS proliferation. However, the exact molecular mechanisms through which FRS2 affects proliferation remain unclear.

FRS2 expression varies across immune-related molecular and immune subtypes in SARC, potentially impacting anti-tumor immunotherapy and survival through immune infiltration. Abnormal FRS2 expression in specific cancer subtypes might not be reflected in broader cancer populations. Our analysis of FRS2's correlation with immune lymphocytes and immunomodulatory factors in SARC suggests that its role in prognosis may differ among molecular or immune subtypes. Future research should focus on FRS2 expression across different cancer subtypes and specific immune profiles to better understand its prognostic value and therapeutic potential.

Recent advancements have shifted perspectives on dendritic cells (DCs), which are crucial for immune activation and antigen presentation. Their role in acquiring and processing antigens for T cell activation has been recognized for decades, culminating in Ralph Steinman's 2011 Nobel Prize for his discovery of DCs 27-29. DC-based immunotherapy is an established strategy to leverage a patient's immune system against metastatic hormone-refractory cancers. Various DC vaccines have demonstrated immunogenicity and some clinical efficacy 30,31. Our study provides evidence that FRS2 may serve as a viable target for adjuvant DC vaccine therapy in SARC. However, there remains a lack of consensus on DC vaccine manufacturing protocols. Ongoing research into the biological mechanisms of DCs' antitumoral and protumoral functions is crucial to fully realize their therapeutic potential 32.

This study has several limitations despite the thorough analysis and validation of FRS2. Firstly, systematic bias is a concern, as reproducibility of data across different laboratories can vary. Additionally, further research is needed to simulate the effects of FRS2 on proliferation and metastasis through animal experiments. Lastly, retroperitoneal liposarcoma is rare, and our patient sample size is relatively small. To address these limitations and obtain more reliable and accurate results, future research will incorporate a larger patient cohort and extended follow-up durations.

## Conclusion

FRS2 is a significant prognostic biomarker in various cancers and a promising therapeutic target for RLPS. It may also influence immune infiltration in the tumor microenvironment. Our study highlights FRS2 as a potential predictor of prognosis in RLPS and supports ongoing preclinical research into fibroblast growth factor receptor inhibitors for RLPS treatment.

## Supplementary Material

Supplementary figure and tables.

## Figures and Tables

**Table 1:** Univariate and multivariate analysis of OS in 82 RLPS patients.

Variable	Univariate			Multivariate		
	HR	95% CI	*P* value	HR	95% CI	*P* value
Gender (Male vs. Female)	1.322	0.749-2.333	0.336			
Age (>60 yr. vs. ≤60 yr.)	0.894	0.498-1.604	0.706			
Tumor size (>15 vs. ≤15 cm)	1.313	0.681-2.531	0.416			
FNCLCC grade (G2/G3 vs. G1)	0.371	0.198-0.694	**0.002**	0.528	0.194-1.438	0.211
Histology (WDLPS vs. DDLPS)	2.359	1.295-4.296	**0.005**	1.442	0.541-3.843	0.465
Multifocality (Yes vs. No)	0.61	0.319-1.167	0.136			
Recurrence (Yes vs. No)	2.157	1.180-3.946	**0.013**	2.048	1.094-3.835	**0.025**
Necrosis (Yes vs. No)	1.125	0.572-2.212	0.733			
FRS2 expression (High vs. Low)	1.915	0.990-3.702	0.053			

Abbreviations: HR: hazard ratio; CI: confidence interval; The bold P value indicated significant difference.

**Table 2:** Univariate and multivariate analysis of disease-free survival in 82 RLPS patients.

Variable	Univariate			Multivariate		
	HR	95% CI	*P* value	HR	95% CI	*P* value
Gender (Male vs. Female)	1.258	0.691-2.292	0.453			
Age (>60 yr. vs. ≤60 yr.)	1.373	0.754-2.498	0.3			
Tumor size (>15 vs. ≤15 cm)	2.21	0.979-4.989	0.056			
FNCLCC grade (G2/G3 vs. G1)	2.743	1.277-6.057	**0.015**	0.89	0.250-3.168	0.857
Histology (WDLPS vs. DDLPS)	0.385	0.218-0.679	**0.004**	0.897	0.279-2.889	0.856
Multifocality (Yes vs. No)	1.13	0.608-2.102	0.699			
Necrosis (Yes vs. No)	1.923	0.981-3.772	0.057			
FRS2 expression (High vs. Low)	3.911	1.707-8.957	**0.001**	4.159	1.800-9.610	**0.001**

Abbreviations: HR: hazard ratio; CI: confidence interval; The bold P value indicated significant difference.

## Abbreviations

RLPS: retroperitoneal liposarcoma
FRS2: Fibroblast growth factor receptor substrate 2
MDM2: Mouse Double Minute 2
STS: soft tissue sarcoma
WDLPS: well-differentiated liposarcoma
DDLPS: dedifferentiated liposarcoma
PLPS: pleomorphic liposarcoma
MLPS: myxoid/round cell liposarcoma
FNCLCC: Federation Nationale des Centers de Lutte Contre le Cancer
IRS: immunoreactivity score
OS: overall survival
DFS: disease-free survival
IHC: immunohistochemistry
PPI: protein-protein interaction
