# Supplementary material for: Oncogenic and Immunological Roles of FRS2 and its Potential Value in Retroperitoneal Liposarcoma: from Bioinformatics Analysis to Clinicopathological Evidence
Source: Int J Med Sci. 2025 Mar 10;22(8):1825–36. doi: 10.7150/ijms.103802 (PMC11983304; doi:10.7150/ijms.103802)
Supplement: Supplementary file 1 — Supplementary figure and tables. [file ijmsv22p1825s1.pdf]

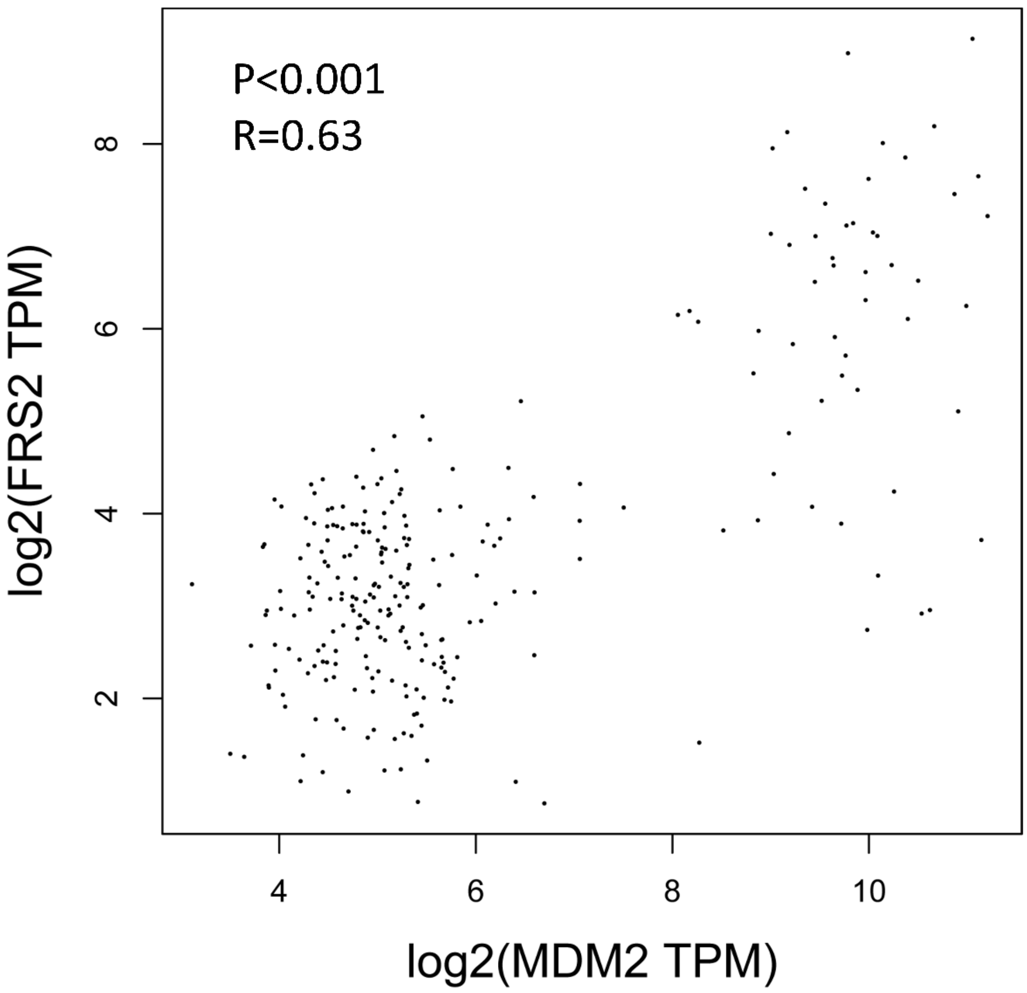

Supplementary Figure 1

GEPIA2 to identify the relationship between MDM2 and FRS2 in SARC.

Supplemental Table 1. 33 cancers in TCGA database in abbreviated form

ACC	Adrenocortical carcinoma
BLCA	Bladder urothelial carcinoma
BRCA	Breast invasive carcinoma
CESC	Cervical squamous cell carcinoma and Endocervical adenocarcinoma
CHOL	Cholangiocarcinoma
COAD	Colon adenocarcinoma
DLBC	Lymphoid neoplasm diffuse large B-cell lymphoma
ESCA	Esophageal carcinoma
GBM	Glioblastoma multiforme
HNSC	Head and neck squamous cell carcinoma
KICH	Kidney chromophobe
KIRC	Kidney renal clear cell carcinoma
KIRP	Kidney Papillary Cell Carcinoma
LAML	Acute myeloid leukemia
LGG	Brain lower grade glioma
LIHC	Liver hepatocellular carcinoma
LUAD	Lung adenocarcinoma
LUSC	Lung squamous cell carcinoma
MESO	Mesothelioma
OV	Ovarian serous cystadenocarcinoma
PAAD	Pancreatic adenocarcinoma
PCPG	Pheochromocytoma and Paraganglioma
PRAD	Prostate adenocarcinoma
READ	Rectum adenocarcinoma
SARC	Sarcoma
SKCM	Skin cutaneous melanoma
STAD	Stomach adenocarcinoma
TGCT	Testicular germ cell tumors
THCA	Thyroid carcinoma
THYM	Thymoma
UCEC	Uterine corpus endometrial carcinoma
UCS	Uterine carcinosarcoma
UVM	Uveal melanoma

Supplemental Table 2. Clinicopathological characteristics of 82 RLPS patients

Clinicopathological Characteristics	N	%
**Gender**		
Male	45	54.88
Female	37	45.12
**Age (years)**		
≤60	43	52.44
>60	39	47.56
**Tumor size (cm)**		
≤15	21	25.61
15-30	47	57.32
>30	14	17.07
**FNCLCC Grade**		
Low (G1)	22	26.83
High (G2/G3)	60	73.17
**Histology**		
DDLPS	56	68.29
WDLPS	26	31.71
**Multifocality**		
No	54	65.85
Yes	28	34.15
**Recurrence**		
No	39	47.56
Yes	43	52.44
**Necrosis**		
No	65	79.27
Yes	17	20.73

Abbreviations: RLPS, retroperitoneal liposarcoma; FNCLCC, Federation Nationale des Centers de Lutte Conter le Cancer; WDLPS, well-differentiated liposarcoma; DDLPS, dedifferentiated liposarcoma.

Supplemental Table 3 Correlations between FRS2 expression and clinicopathological characteristics in RLPS patients.

Characteristics	Cases	FRS2 expression				*P* value
		High (%)		Low (%)		
**Gender**						0.132
Male	45	23	51.11%	22	48.89%	
Female	37	25	67.57%	12	32.43%	
**Age**						**0.009**
≤60	43	31	72.09%	12	27.91%	
>60	39	17	43.59%	22	56.41%	
**Tumor size**						0.185
≤15	21	10	47.62%	11	52.38%	
15-30	47	27	57.45%	20	42.55%	
>30	14	11	78.57%	3	21.43%	
**FNCLCC Grade**						0.570
Low (G1)	22	14	63.64%	8	36.36%	
High (G2/G3)	60	34	56.67%	26	43.33%	
**Histology**						0.707
DDLPS	56	32	57.14%	24	42.86%	
WDLPS	26	16	61.54%	10	38.46%	
**Multifocality**						0.773
No	54	31	57.41%	23	42.59%	
Yes	28	17	60.71%	11	39.29%	
**Recurrence**						0.939
No	39	23	58.97%	16	41.03%	
Yes	43	25	58.14%	18	41.86%	
**Necrosis**						0.568
No	65	38	58.46%	27	41.54%	
Yes	17	10	58.82%	7	41.18%	

Abbreviations: FRS2, Factor Receptor Substrate 2; The bold P value indicated significant difference.
